# Initiation and Characterization of Small Cell Lung Cancer Patient-Derived Xenografts from Ultrasound-Guided Transbronchial Needle Aspirates

**DOI:** 10.1371/journal.pone.0125255

**Published:** 2015-05-08

**Authors:** Wade C. Anderson, Michael B. Boyd, Jorge Aguilar, Brett Pickell, Amy Laysang, Marybeth A. Pysz, Sheila Bheddah, Johanna Ramoth, Brian C. Slingerland, Scott J. Dylla, Edmundo R. Rubio

**Affiliations:** 1 Virginia Tech Carilion School of Medicine, Section of Pulmonary, Critical Care, Environmental, and Sleep Medicine, Carilion Clinic, Roanoke, Virginia, United States of America; 2 Stemcentrx, Inc., South San Francisco, California, United States of America; University of Colorado Denver, UNITED STATES

## Abstract

Small cell lung cancer (SCLC) is a devastating disease with limited treatment options. Due to its early metastatic nature and rapid growth, surgical resection is rare. Standard of care treatment regimens remain largely unchanged since the 1980’s, and five-year survival lingers near 5%. Patient-derived xenograft (PDX) models have been established for other tumor types, amplifying material for research and serving as models for preclinical experimentation; however, limited availability of primary tissue has curtailed development of these models for SCLC. The objective of this study was to establish PDX models from commonly collected fine needle aspirate biopsies of primary SCLC tumors, and to assess their utility as research models of primary SCLC tumors. These transbronchial needle aspirates efficiently engrafted as xenografts, and tumor histomorphology was similar to primary tumors. Resulting tumors were further characterized by H&E and immunohistochemistry, cryopreserved, and used to propagate tumor-bearing mice for the evaluation of standard of care chemotherapy regimens, to assess their utility as models for tumors in SCLC patients. When treated with Cisplatin and Etoposide, tumor-bearing mice responded similarly to patients from whom the tumors originated. Here, we demonstrate that PDX tumor models can be efficiently established from primary SCLC transbronchial needle aspirates, even after overnight shipping, and that resulting xenograft tumors are similar to matched primary tumors in cancer patients by both histology and chemo-sensitivity. This method enables physicians at non-research institutions to collaboratively contribute to the rapid establishment of extensive PDX collections of SCLC, enabling experimentation with clinically relevant tissues and development of improved therapies for SCLC patients.

## Introduction

Lung cancers are the leading cause of cancer-related deaths worldwide. Among these, small cell lung cancer (SCLC) has the highest mortality rate, accounting for ~15% of lung cancer deaths in the United States[1]. Five-year survival for SCLC patients remains near 5%, with most succumbing to disease within one year of diagnosis. SCLC is highly metastatic and progresses quickly. Surgical resection does not improve survival and, therefore, is rarely prescribed. Hence, chemotherapy remains the first line of treatment[2].

The most commonly administered chemotherapeutic regimen for SCLC is Cisplatin and Etoposide (P/E), as it has been for over three decades[2]. This regimen achieves significant initial response in most patients, often reducing tumors to undetectable levels. Unfortunately, tumors nearly always recur shortly after cessation of treatment. Recurrent tumors are generally more aggressive and resistant to subsequent attempts to slow tumor growth using P/E or other approved chemotherapeutic regimens, such as Topotecan[3–5]. In even less fortunate patients, first line P/E treatment produces a partial response, accelerating the need for second line therapy when tolerated. Although current chemotherapeutic regimens extend survival for SCLC patients (versus no treatment), the benefits are limited and the 5-year survival impact is trivial. One explanation for the negligible progress in the discovery and development of novel therapeutics that impact SCLC patient survival is the lack of appropriate tumor models with which to study the disease. Proper models should enable preclinical evaluation of investigational therapies, morphologically resemble patient tumors, and respond similarly to standard chemotherapeutic regimens *in vivo*.

Patient-derived xenograft (PDX) models, which are generated from freshly resected tumor tissue implanted, grown, and exclusively passaged in severely immunocompromised mice, are increasingly being generated and utilized to study human tumor biology[6–8]. These models retain significant similarities to the patient tumors from which they were generated. PDX tumor models generated from various other tumor types generally retain their sensitivity profile to standard of care chemotherapy responses observed clinically. In addition to facilitating the growth of human tumors for *ex vivo* study, PDX tumors also offer physiologically relevant models with which to study tumor biology and evaluate preclinical efficacy of therapeutic agents *in vivo*. PDX tumor models can also be cryopreserved and thoroughly studied over time without extensive passaging, preventing the adverse results of prolonged *in vitro* culture, such as the genomic alterations that plague both cell lines and their resulting conventional cell line xenografts[9, 10].

Because SCLC is rarely surgically excised, research in this indication has not benefited from the availability of PDX tumor models generated from resections, though some SCLC PDX models do exist[10–13]. Access to primary SCLC tumor material is predominantly limited to diagnostic fine needle aspirations which are commonly considered too small to facilitate engraftment. Leong et al. have demonstrated that these fine needle aspirations can generate PDX tumors morphologically and genetically similar to their matched primary tumors[11]. These fine needle aspirations are frequently collected at clinics and hospitals without onsite access to immunocompromised animals or the infrastructure to generate new PDX lines. Here, we show that single, primary SCLC fine needle aspiration specimens, taken subsequent to the collection of diagnostic samples and shipped overnight to a collaborating research facility, are able to efficiently establish PDX tumors morphologically similar to the primary tumor from which they originated. Furthermore, we demonstrate that *in vivo* chemo-responsiveness of these PDX tumors to treatment with combined P/E is generally conserved from the primary tumors to their corresponding PDX tumors in mice. This work demonstrates that researchers and clinicians, working collaboratively even across great distances, can quickly and efficiently establish large collections of PDX tumor lines from abundantly available fine needle aspirates of SCLC. Conserved histomorphology and responsiveness to standard of care chemotherapeutic regimens make these excellent models with which to better study SCLC tumor biology, and facilitate the discovery and development of therapeutics that improve patient survival.

## Materials and Methods

### Patients

The study was approved by the Carilion Clinic Institutional Review Board. All 12 patients with small cell lung cancer gave written informed consent before participating in the study. Samples were taken by endobronchial ultrasound-guided transbronchial needle aspiration (EBUS-TBNA), either from a central primary tumor or from suspected nodal involvement. Staging classification was made according to both the Veterans Administration Lung Study Group and International Association for the Study of Lung Cancer staging systems[14, 15]. Performance status was determined at the time of initial diagnosis[16].Patients were offered standard of care treatment and outcome measurements were monitored.

### EBUS-TBNA

All procedures were performed under deep sedation using a flexible ultrasound bronchoscope (CP-EBUS XBF-UC260F, Olympus, Tokyo, Japan)[17] and a 21G Vizashot needle. At least 6 transbronchial needle biopsy samples were taken at each suspected tumor or nodal station. The initial samples were processed in a standard institutional fashion, with preparation of slides for rapid on-site cytological interpretation, as well as collecting material to build a cell—block for delayed pathological evaluation. The last sample collected was expelled into ice-cold HypoThermasol-FRS (Sigma, St. Louis, MO) in a 1.5 mL microcentrifuge tube containing a volume at least 10-fold greater than the volume of the tissue, and shipped overnight to Stemcentrx, Inc. for implantation within 30 hours of biopsy.

### Clinical treatment regimen of SCLC donor patients post-biopsy

Eight of twelve patients from whom tumor specimens were biopsied received intravenous 60–80 mg/m^2^ Cisplatin on day 1 plus 80–120 mg/m^2^ Etoposide on days 1–3 every 21–28 days with an anticipation of at least four cycles of therapy. Both regimens required hydration and administration of antiemetic drugs. If leukocyte counts fell below 2,000 per mm^3^, or neutrophil counts fell below 1000 per mm^3^, recombinant human granulocyte colony-stimulating factor was administered until leukocyte and/or neutrophil counts were restored. Dose adjustments were made based upon identification of chemotherapy-associated organ toxicities. Three patients opted out of treatment. One patient was lost to follow-up and thus their treatment course is unknown.

The following criteria were used to assess patient responses to standard of care treatment with Cisplatin and Etoposide (P/E). Radiological imaging, either Computed Chest or Positron Emission Tomography, was utilized to determine response to standard of care therapy. Response Evaluation Criteria In Solid Tumors (RECIST) was utilized to grade tumor response as follows: Complete Response (CR): Disappearance of all target lesions. Partial Response (PR): At least a 30% decrease in the sum of the longest diameter (LD) of target lesions, taking as reference the baseline sum LD. Stable Disease (SD): Neither sufficient shrinkage to qualify for PR nor sufficient increase to qualify for Progressive Disease (PD), taking as reference the smallest sum LD since the treatment started. PD: At least a 20% increase in the sum of the LD of target lesions, taking as reference the smallest sum LD recorded since the treatment started or the appearance of one or more new lesions[18]. Overall Survival (OS) was defined as the interval between initial diagnosis and patient death measured in weeks.

### PDX generation

This study was approved by the Stemcentrx Institutional Animal Care and Use Committee (Protocols SCAR-3-2008 and SCAR-5-2008). Mouse studies were performed in accordance with American Association for Laboratory Animal Science. All surgery was performed under Isoflurane anesthesia, and all efforts were made to reduce animal suffering. Mice were euthanized using compressed CO_2_ gas, according to the most recent AVMA Guidelines on Euthanasia. Upon receipt by Stemcentrx, each SCLC tumor biopsy specimen was pelleted by light centrifugation (50 rcf for 5 minutes), re-suspended in 100–150μL Medium-199 (Mediatech, Inc., Manassas, VA) and mixed with an equal volume of Matrigel (BD Biosciences, Milpitas, CA). Then, 100μL of the solution was subcutaneously injected under the lower mammary fat pads of 8–10 week old female NOD/SCID recipient mice. Mouse health, weight, and tumor volume(s) were assessed, at least, weekly. When tumor volumes measured between 800–1,500 mm^3^, recipients were humanely euthanized and tumors were resected.

### Propagation and analysis of PDX tumors

Freshly resected tumors were dissociated to a single cell suspension as described previously[19, 20]. At each passage of tumor propagation, human epithelial origin was confirmed by positive staining by flow cytometry using anti-human EpCAM (Clone 9C4), and negative staining with anti-human CD45 (Clone HI30), anti-mouse CD45 (Clone 30-F11), and anti-mouse H-2K^d^ (Clone SF1.1) antibodies (all from BioLegend, San Diego, CA). All flow cytometry antibodies were used at a final concentration of 10 μg/mL. To propagate the PDX tumors, dissociated cells were suspended 1:1 by volume in Matrigel at a concentration of 50,000 cells per 100 μL final volume per injection site. Cells were bilaterally implanted under the lower mammary fat pads using a 27G needle and tumor growth was monitored weekly.

Time to Progression (TTP) was defined as the interval between treatment responses and identification of progressive disease by RECIST measured in weeks. For measuring patient-derived xenograft tumor responses to standard of care treatment with Cisplatin and Etoposide, the following criteria were used: Doubling Time was defined as the mean time period wherein tumor volume doubled (days); The rate of tumor volume growth was monitored, calculated using the formula d_dbl_ = (d_i_-d_f_)/log_2_(v_i_-v_f_) and averaged for all tumors while in the range of 150mm^3^—1200mm^3^; Percentage Tumor Growth Inhibition (%TGI) was calculated as the average volume of chemo-treated tumors divided by the average volume of vehicle-treated tumors 21 days after initial treatment.

Primary SCLC biopsies and PDX tumors were formalin fixed and paraffin embedded (FFPE), and planar sections of tissue blocks were cut and mounted on glass microscope slides. For IHC, xylene de-paraffinized tissue sections were pretreated with Antigen Retrieval Solution (Dako, Carpinteria, CA), blocked with 10% donkey serum in 3% BSA in PBS buffer, and then incubated with primary antibody. Primary antibodies used were as follows: Synaptophysin (1:200; Clone SP11; Spring Bioscience, Pleasanton, CA), Chromogranin-A (0.2 μg/mL; Clone SP12; Spring Bioscience, Pleasanton, CA), and CD56 (0.2 μg/mL; Clone EP2567Y; Origene, Rockville, MD), Keratin 5 (1.4 μg/mL; Clone SP178, Spring Bioscience, Pleasanton, CA), Keratin 6 (0.15 μg/mL; Clone SP87, Spring Bioscience, Pleasanton, CA), Keratin 7 (0.7 μg/mL; Clone SP52, Spring Bioscience, Pleasanton, CA), Keratin 14 (1 μg/mL; Clone LL002, Abcam, Cambridge, MA), Keratin 20 (1:50; Clone SPM140, Abcam, Cambridge, MA), TTF1 (Clone 8G7G3/1, Biocare Medical, Concord, CA), TP63 (0.125 μg/mL; Clone 4A4, Biocare Medical, Concord, CA), Napsin A (Clone TMU-Ad02, Biocare Medical, Concord, CA). Sections were incubated in biotin-conjugated, species-specific secondary antibodies (Immunoresearch, West Grove, PA) followed by incubation in streptavidin-HRP (ABC Elite Kit; Vector Labs, Burlingame, CA). Chromogenic detection was developed with 3, 3’-diaminobenzadine (Thermo Scientific, Rockford, IL) and tissues were counterstained with hematoxylin and imaged with a light microscope with 40X magnification.

For IHC staining, the following negative control antibodies and positive control tissues were used. CHGA: Mouse IgG1 (BioLegend, Inc., San Diego, CA) and normal human pancreas. Synaptophysin: Rabbit IgG (Jackson Immunoresearch, West Grove, PA) and normal human pancreas. CD56: Rabbit IgG (Jackson Immunoresearch, West Grove, PA) and normal human pancreas. Keratin 5: Rabbit IgG (Jackson Immunoresearch, West Grove, PA) and normal human prostate. Keratin 6: Rabbit IgG (Jackson Immunoresearch, West Grove, PA) and normal human prostate. Keratin 7: Rabbit IgG (Jackson Immunoresearch, West Grove, PA) and normal human prostate. Keratin 14: Mouse IgG1 (BioLegend, Inc., San Diego, CA) and normal human skin. Keratin 20: Mouse IgG2a (BioLegend, Inc., San Diego, CA) and normal human colon. Napsin A: Rabbit IgG (Jackson Immunoresearch, West Grove, PA) and normal human lung. P63: Mouse IgG2a (BioLegend, Inc., San Diego, CA) and normal human prostate. TTF-1: Mouse IgG1 (BioLegend, Inc., San Diego, CA) and normal human lung.

## Results

### Safety of minimally invasive sampling technique

From each of the 12 consenting SCLC patients who participated in this study, a single additional needle biopsy sample was collected by EBUS-TBNA for the purpose of attempting to initiate xenograft tumors. These cell aspirate samples were drawn from either primary lung tumors, or involved nodes from a range of patients averaging 62 years old (Table 1). Ten of these twelve tumors were diagnosed as metastatic (*i*.*e*. extensive disease) and, of those, two samples were collected from involved lymph nodes. No patients experienced adverse effects attributable to the additional biopsy sample collection.

**Table 1:** SCLC Tumor Donor Characteristics.

Patient	VA Stage	AJCC Stage	Resection Site	Time to 150mm^3^ (d)	TTP (wks)	Survival (wks)
LU064	EXT	(T2a,N2, M1b) IV	Lung	116	60	79
LU073	LIM	(T2b, N2, M0) IIIA	Lung	151	31	76
LU080	EXT	(T2a,N3, M1b) IV	Node	81	9	35
LU086	EXT	(T4, N2, M1b) IV	Lung	95	7	40
LU095	EXT	(T3, N2, M1b)IV	Lung	81	9	102
LU108	EXT	(T2b,N2,M1b) IV	Lung	NA	NA	3
LU112	EXT	(T2b,N2,M1b) IV	Lung	NA	NA	2
LU117	EXT	(T4 N2 M1b) IV	Lung	82	Lost	Lost
LU122	LIM	(T4,N2 M0) IIIB	Lung	NA	20	Alive
LU124	EXT	(T4, N3 M0) IIIB	Lung	112	Remission	Alive
LU125	EXT	(T4,N3, M1a) IV	Lung	NA	NA	1
LU129	EXT	(T3, N3, M1b) IV	Node	110	7	25

### PDX tumor generation and characterization

After collection, specimens were shipped overnight on ice from Carilion Clinic Hospital in Roanoke, Virginia to Stemcentrx in San Francisco, California. Within 30 hours of biopsy, cells from the aspiration samples were subcutaneously implanted into immunocompromised NOD/SCID recipient mice and monitored for tumor growth for up to 32 weeks. While a single cell aspirate sample may not fully represent the cellular diversity of a patient tumor, more thorough and representative sampling of the same patient tumor would have unnecessarily increased risk to the patients. By 26 weeks post-implantation, 8 of 12 (67%) patient samples produced confirmed SCLC tumors, with the average time to reach 150 mm^3^ ranging from 81–151 days post-transplant. To facilitate both immediate and prospective studies, resulting “passage 1” (p1) tumors were fixed in formalin, or dissociated into single cell suspensions for immediate propagation into new recipient mice, further phenotypic and/or genetic characterization, or cryopreservation. Among the 4 SCLC patient samples that did not result in useful SCLC PDX tumor lines, three failed to initiate tumors (LU108, LU112, LU125) and one (LU122) consisted of an outgrowth of human B-cell lymphoma. To ensure faithful propagation of uncontaminated tumors, a SNP fingerprinting assay was performed on primary tumor cells and PDX cells at each passage. These results confirm that each PDX tumor is unique and derived from its cognate primary tumor.

**Fig 1:**
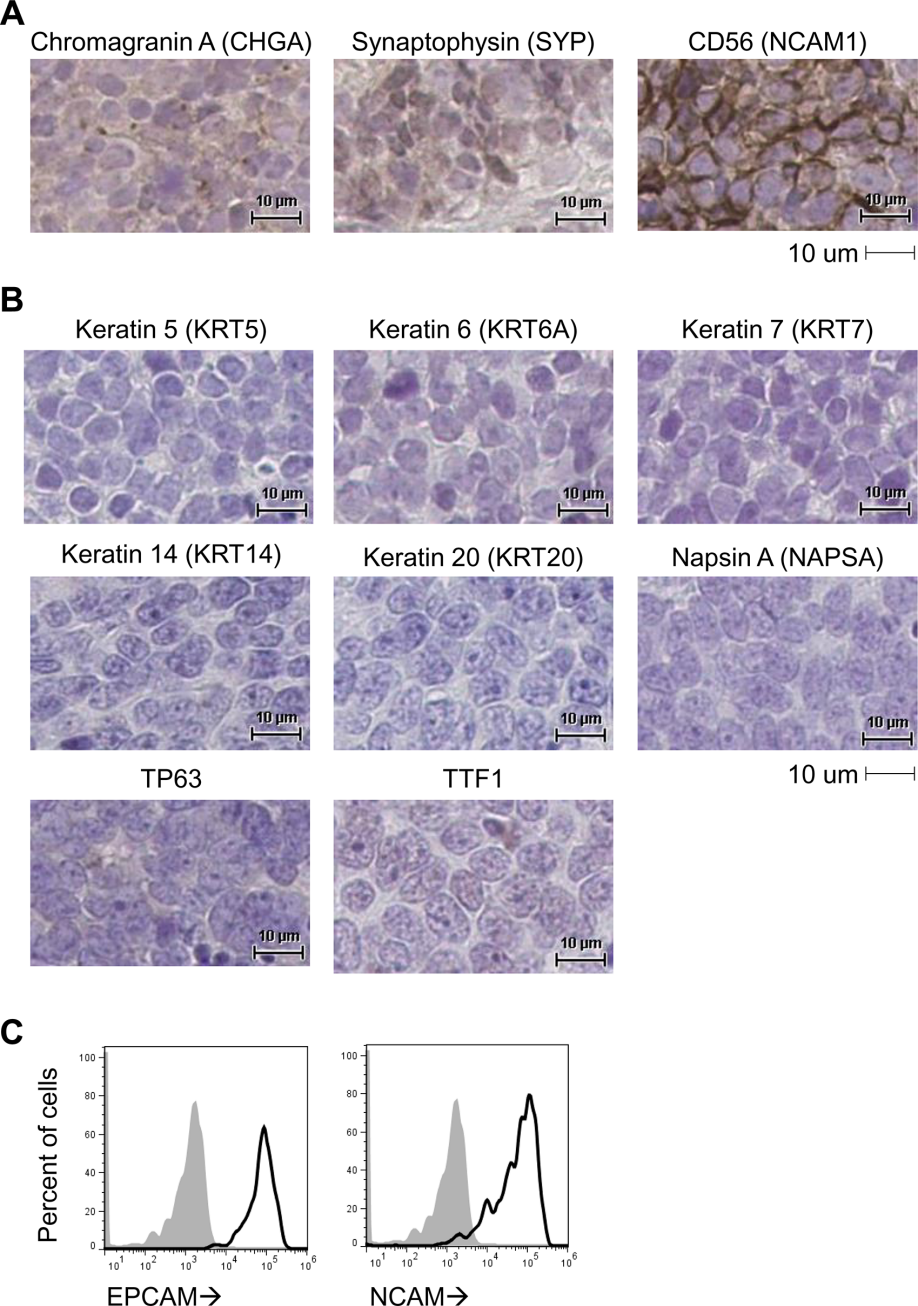
Expression of SCLC antigens is maintained in PDX tumor models. FFPE sections were prepared from PDX tumors (LU086p1 is represented). (A) Tissue sections were stained by IHC for diagnostic SCLC markers Chromagranin A (CHGA), Synaptophysin (SYP), or CD56. Scale bars represent 10um. (B) Tissue sections were stained by IHC for diagnostic non-SCLC markers Keratin 5 (KRT5), Keratin 6 (KRT6A), Keratin 7 (KRT7), Keratin 14 (KRT14), Keratin 20 (KRT20), Napsin A (NAPSA), TP63, or TTF1. Scale bars represent 10um. (C) PDX tumor cells were dissociated into single-cell suspensions and analyzed by flow cytometry for expression of EpCAM (CD326) and NCAM1 (CD56). Histograms displaying expression levels are shown (dark black line), whereas background signal was determined using a matched isotype control antibody (filled gray).

SCLC tumors have a distinct morphology characterized by the flatness of cells, nuclear moulding, and a sparse cytoplasm[21]. In most cases, these features can be used to clearly distinguish SCLC from the various forms of non-small cell lung cancer (NSCLC). SCLC tumors have variable morphology, ranging from high cellularity to scarce tumor cells amid stroma and necrotic tissue. Nevertheless, the hallmark oat cell morphology, nuclear moulding, and scanty cytoplasm can be readily confirmed in PDX tumor samples (Fig 1A and 1B, S1 and S2 Figs). Cytological smears of cell aspirates from patient tumors and FFPE sections from matched PDX tumors were stained with Kwik-Diff stain. Morphological similarities were observed between primary and xenograft tumors (S5 Fig). While cytological smears from cell aspirates poorly preserve tumor tissue organization, cellular morphologies are similar.

To confirm their SCLC identities, tumors were assessed for their expression of several hallmark proteins commonly used to confirm diagnosis. First, FFPE tissue sections from PDX tumors were stained by IHC for the positive SCLC diagnostic antigens Chromogranin-A, Synaptophysin, and CD56 (Fig 1A and S1 Fig). Further, PDX tissue sections generally did not express antigens characteristic of non-small cell lung cancers, including Keratin 5, Keratin 6, Keratin 7, Keratin 14, Keratin 20, TTF1, TP63, and Napsin A (Fig 1B and S2 Fig). Positive staining for Keratin 5, Keratin 6, Keratin 14, or TP63 can indicate squamous cell carcinomas of the lung. Positive staining for Keratin 7, TTF1, or Napsin A can indicate lung adenocarcinoma. Keratin 20 is typically expressed in cancers of the gastrointestinal tract and its negative expression can be used to exclude cancers of nonpulmonary origin. Only 3 of 8 SCLC PDX in this study were positive for TTF-1 expression. While TTF-1 expression in SCLC is common, it is primarily used to identify pulmonary adenocarcinoma and the positive staining is likely influenced by antibody clone. Finally, tumors were dissociated to single-cell suspensions and characterized by flow cytometry to verify their human epithelial origin (*i*.*e*. human EpCAM+) and confirm CD56 expression (Fig 1C and S3 Fig). Representative IHC and flow cytometry images are shown for LU086 in Fig 1 and a summary of IHC staining for all tested PDX lines is shown in Table 2.

**Table 2:** Immunohistochemical Staining of Diagnostic Lung Cancer Markers.

Lung Tumor	CHGA	SYP	NCAM1	KRT5	KRT6A	KRT7	KRT14	KRT20	NAPSA	TP63	TTF1
LU064p1	+	-	+	-	-	-	-	-	-	-	+
LU073p1	+	-	+	-	-	+	-	-	-	-	-
LU080p2	-	+	+	-	-	-	-	-	-	+	<5%
LU086p1	+	-	+	-	-	-	-	-	-	-	-
LU095p1	+	-	+	-	<5%	-	-	-	-	-	-
LU117p2	+	+	+	-	-	<5%	-	-	-	-	-
LU124p4	+	+	-	<5%	<5%	-	-	+	-	-	-
LU129p2	+	+	+	-	-	-	-	-	-	-	+

On PDX tumors, we also performed targeted resequencing of oncogenes (S1 Table). TP53 is the most commonly mutated gene in SCLC, affecting roughly 3 of 4 tumors. We found TP53 mutations in 6 of 8 tumors. RB1 is mutated in approximately half of SCLC and we found RB1 mutations in 3 of our 8 tested SCLC PDX tumors. These mutation frequencies are consistent with reported frequencies for SCLC.

In summary, SCLC PDX tumors retain striking resemblance to their primary SCLC tumors despite having been initiated from very little material. Furthermore, the efficiency with which PDX tumors can be established using needle biopsy material suggests that the tumor perpetuating cell (TPC; *i*.*e*. cancer stem cell) frequency is relatively high, which might be expected given the aggressiveness and refractory nature of SCLC.

### PDX tumor response to P/E reflects clinical response

**Fig 2:**
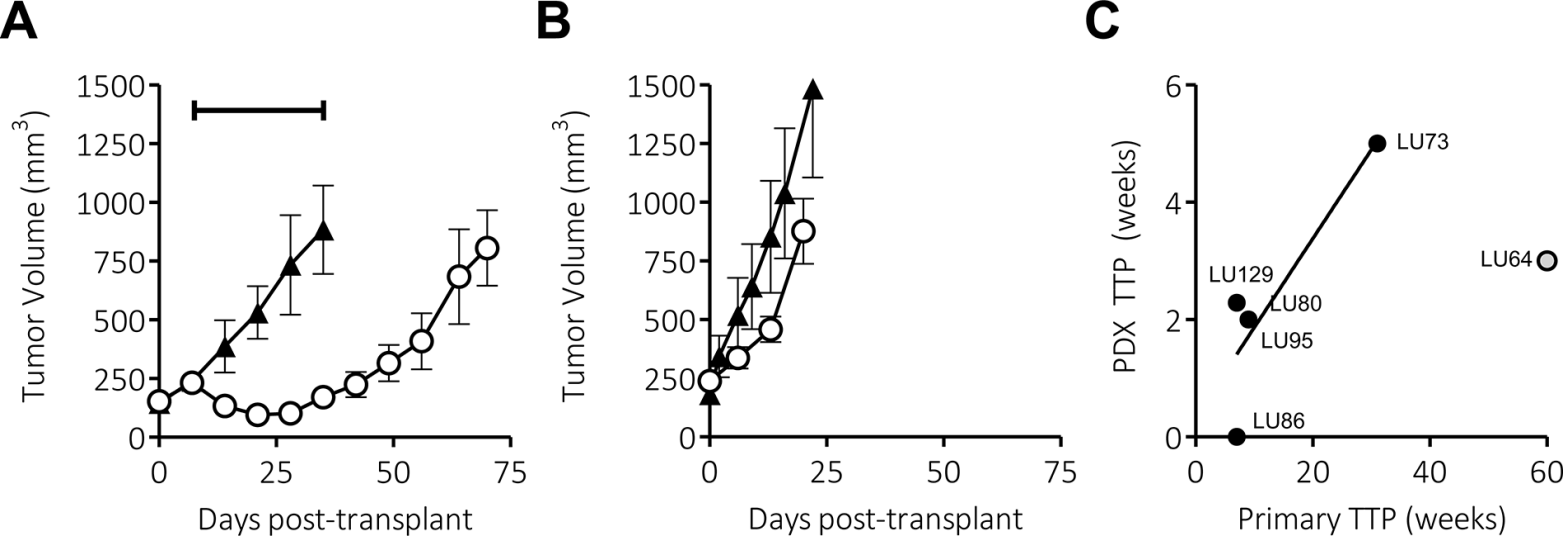
PDX response to P/E *in vivo* generally reflects clinical response. Upon reaching a mean tumor volume of 150–200 mm^3^, mice bearing A) LU073p2 or B) LU086p3 PDX tumors were randomized, administered either vehicle (closed triangles) or P/E (open circles; 5 mg/kg Cisplatin on day 1 and 8 mg/kg Etoposide on days 1, 2 & 3 of treatment), and tumors were measured weekly. The bracket indicates the time to progression (TTP). C) The mean TTP of PDX tumors following one course of P/E treatment was plotted versus observed clinical TTP. Data is represented as Mean ± SEM and reflects cohorts of n = 5 mice per group.

To determine whether SCLC PDX tumors, established as described herein, retain the P/E response characteristics observed in patients from whom the PDX tumors were generated, PDX tumors were exposed to combined P/E regimens at near maximum tolerated doses that equate to similar doses to the regimen used in patients (*i*.*e*. 5 mg/kg Cisplatin & 24 mg/kg Etoposide, which equates to roughly 20 mg/m^2^ Cisplatin and 94 mg/m^2^ Etoposide). Following subcutaneous implantation of SCLC PDX tumor cells and randomization into cohorts of 5–8 mice per group once tumors reached 150 mm^3^–200 mm^3^, mice were dosed on the day of randomization with 5 mg/kg Cisplatin and 8 mg/kg Etoposide, and again the on following two days with an additional 8 mg/kg Etoposide each day. Cohorts receiving the vehicle were administered 0.9% NaCl in the same volumes used to dose mice with P/E. Most SCLC PDX tumors responded to this single course of P/E chemotherapy, resulting in a mean tumor growth inhibition of 79 ± 17% versus vehicle-treated mice (Table 3). Despite a robust response by most PDX tumors, as is observed in humans, there was invariable tumor recurrence, generally observed within 3 weeks of randomization (17 ± 10 days; n = 8), with all SCLC PDX tumors recurring within 5 weeks. For example, LU073 PDX tumors responded well to P/E, demonstrating an 82% tumor growth inhibition and 35 day (*i*.*e*. 5 weeks) time to progression (Fig 2A and Table 3), whereas the patient from whom LU073 PDX tumors were established exhibited a 31 week time to progression in the clinic following a full course of therapy (Table 1). By contrast, the LU086 PDX tumor model initiated from an extensive stage patient who did not respond well to the clinical regimen of P/E (clinical TTP = 7 weeks) was also minimally responsive to therapy with P/E (41% TGI & TTP = 0; Fig 2B). In summary, the vast majority of established SCLC PDX tumor models demonstrated tumor responses that correlated with TTP metrics observed clinically (Fig 2C; n = 5; R-square = 0.77; *P*-value = 0.050). The lone outlier to this trend was PDX LU064 (Fig 2C; grey open circle). The general concordance between primary patient tumor and PDX tumor response to P/E *in vivo* supports the conclusion that SCLC PDX tumors initiated from tumor biopsies reflect patient tumor biology and serve as excellent models with which to better study and understand this aggressive malignancy.

**Table 3:** PDX Growth & P/E Responsiveness.

			Response to P/E SOC	
	Doubling Time (d)	Time to 150mm^3^ (d)	%TGI	TTP (d)
LU064	15.6	60	92%	21
LU073	18.0	81	82%	35
LU080	13.3	41	80%	14
LU086	11.2	59	41%	0
LU095	18.5	75	73%	14
LU117	14.4	38	99%	21
LU124	17.4	62	82%	14
LU129	33.3	69	84%	16
	18 ± 7	61 ± 15	79 ± 17%	17 ± 10

## Discussion

Small cell lung cancer is a very deadly disease, against which little progress has been made in decades. In part, the lack of progress is traceable to a paucity of available highly relevant tumor models for research, stemming from a perceived scarcity of primary tissue suitable for establishing patient-derived xenograft tumors in mice. SCLC is routinely diagnosed through the collection of biopsy samples by TBNA. The advent of EBUS guidance facilitates safer, easier and more accurate biopsies, enabling increased availability of this material for research purposes. Here, we show that small tissue samples from these biopsies are enough to efficiently engraft and establish xenograft tumor lines in mice with minimal risk to the donor patients. These PDX tumors can be generated by distant collaborators and their therapeutic responses mimic those of their matched primary tumors.

The efficiency of engrafting primary tumor specimens in immunocompromised mice is influenced by many factors, including tissue origin, viability, tumor type, stage and aggressiveness, recipient mouse strain, and other technical and environmental factors[6–8]. For example, primary breast tumors have been reported to engraft as xenografts with 12.5% efficiency, whereas NSCLC xenografts have reported engraftment efficiencies as high as 90%[22–24]. Here, we demonstrate that SCLC xenograft tumors can be established with high efficiency, maintain parental tumor histomorphology, and largely replicate the parental tumor response to P/E chemotherapy.

While SCLC PDX tumor lines have been generated and described previously[9–13], the number of available SCLC PDX tumor models remains extremely limited, and the wider generation of these models has not been pursued due to the perceived inability to initiate xenografts from needle biopsy material. In this study, a small collaborative effort originating at a single clinic produced 8 successful SCLC PDX tumor lines in only 19 months without a requirement for local access to immunocompromised animals. Indeed, overnight shipping of SCLC fine needle aspirate specimens appeared to have had a minimal impact on tumor engraftment efficiency, although even better engraftment efficiency might be expected with same-day xenotransplantation. The prodigious success of this study, rapidly establishing a large collection of SCLC PDX tumor lines from a diverse array of donor patients using EBUS-TBNA specimens, demonstrates that this approach can be successfully executed.

Because PDX tumor models were generated from a single needle biopsy from either primary or nodal sites, it is possible that resulting PDX lines do not contain the complete clonal heterogeneity present in patients. Nevertheless, 5 of 6 (83%) PDX lines for which both clinical and PDX response data to P/E treatment regimens were known had correlating responses *in vivo*. The concordance of this data suggests that either few clones were present in SCLC patients at the time of biopsy or a single biopsy pass was able to obtain and confer a representation of the clones present. The lone exception to this observation was LU064, which appeared to have an impaired response to P/E in mice versus the robust response observed in the patient from which it was derived. This discrepancy may reflect the fact that a more aggressive subclone was established in mice or additional mutations may have accrued following implantation.

SCLC tumor models that accurately reflect human disease have been difficult to come by. Traditional cell lines are routinely propagated and studied in an *in vitro* setting; however, these lines have diverse irrevocable discrepancies compared to tumors as they exist in patients[9, 25–27]. Surprisingly, many of the most commonly utilized cell lines with which to study SCLC tumor biology were generated more than 30 years ago[28], and have significant genomic abnormalities likely associated with decades of *in vitro* culture in non-physiological conditions[29]. Daniel *et al*. recently demonstrated that even brief periods of *in vitro* culture irreversibly alters gene expression in SCLC tumor cells[9], thus cell lines extensively expanded *in vitro* are unlikely to appropriately reflect the parental tumor. One might argue that these traditional SCLC cell lines have provided little insight into SCLC tumor biology, as the standard of care has not improved over the same time period that these cell lines have been widely studied. In contrast, SCLC PDX tumor models consisting of tumor cells minimally passaged *in vivo*, never touch a plastic dish, and appear to replicate *in vivo* tumor histomorphology and chemo-responsiveness. SCLC PDX tumors thus present new opportunities for researchers to explore mechanisms underlying tumorigenicity and chemo-resistance.

The 3 tumor specimens that did not result in any growth were each collected from patients who ultimately survived less than a month after sample collection; 2 of which, unique to this study, had Eastern Cooperative Oncology Group (ECOG) scores of 4. It does appear that PDX tumor lines may be most efficiently established from patients who are not at immediate risk of mortality. Each of the 3 “non-engrafting” tumor specimens in this study originated from patients who survived less than 4 weeks following extraction of needle biopsies, while the other 6 patients for which survival data is available had “engrafting” tumors and survived at least 25 weeks after diagnosis. Although this may be counterintuitive given that more advanced tumors might be considered to be more aggressive, the declining systemic health of patients with higher ECOG scores may be detrimental to tumor cell viability due to factors such as hypoxia and/or decreased access to other nutrients, making collection of viable patient tumor tissue difficult. Nevertheless, based on this discussion, it is foreseeable that a clear understanding of the morphology of tumors/adenopathy under EBUS imaging may allow more targeted biopsies to areas of less necrotic material, that may have a better opportunity to generate adequate PDX tumor models. Such theory does require further testing and this is important as we attempt to also generate PDX tumor models to better understand these patients with apparently more aggressive disease.

We have further demonstrated that PDX models can be generated from both limited and extensive stage SCLC patients using small amounts of tissue obtained by EBUS-TBNA. Further, we show that efficient PDX engraftment can be achieved even after overnight storage and shipping of the primary tumor specimen. This enables clinicians with abundant access to primary tumor biopsy material, but without convenient access to research animals, to collaborate with distant researchers to quickly establish SCLC PDX tumor lines. In generating many PDX lines from an array of SCLC patients, it will likely be possible to identify and better characterize subtypes of SCLC, and mine the underlying genetic and proteomic data that distinguish these subtypes and their differential response to therapy. Not only can SCLC PDX tumor models be used to better understand SCLC patient diversity and tumor biology, but they also provide more biologically relevant models for preclinical drug discovery and development. More widespread establishment and use of such models will greatly benefit SCLC research and, ultimately, patient outcomes.

## Supporting Information

S1 Fig PDX Tumors Express Diagnostic SCLC Markers. (TIF)

S2 Fig PDX Tumors Do Not Express Diagnostic Non-SCLC Markers. (TIF)

S3 Fig PDX Tumors Express EpCAM and NCAM. (TIF)

S4 Fig PDX Tumors Respond to Treatment with Cisplatin and Etoposide. (TIF)

S5 Fig Morphology of Primary and Xenograft Tumors. (TIF)

S1 Table Oncogene Mutations. (JPG)
