# Supplementary figures and images for: Initiation and Characterization of Small Cell Lung Cancer Patient-Derived Xenografts from Ultrasound-Guided Transbronchial Needle Aspirates
Source: PLoS One. 2015 May 8;10(5):e0125255. doi: 10.1371/journal.pone.0125255 (PMC4425530; doi:10.1371/journal.pone.0125255)

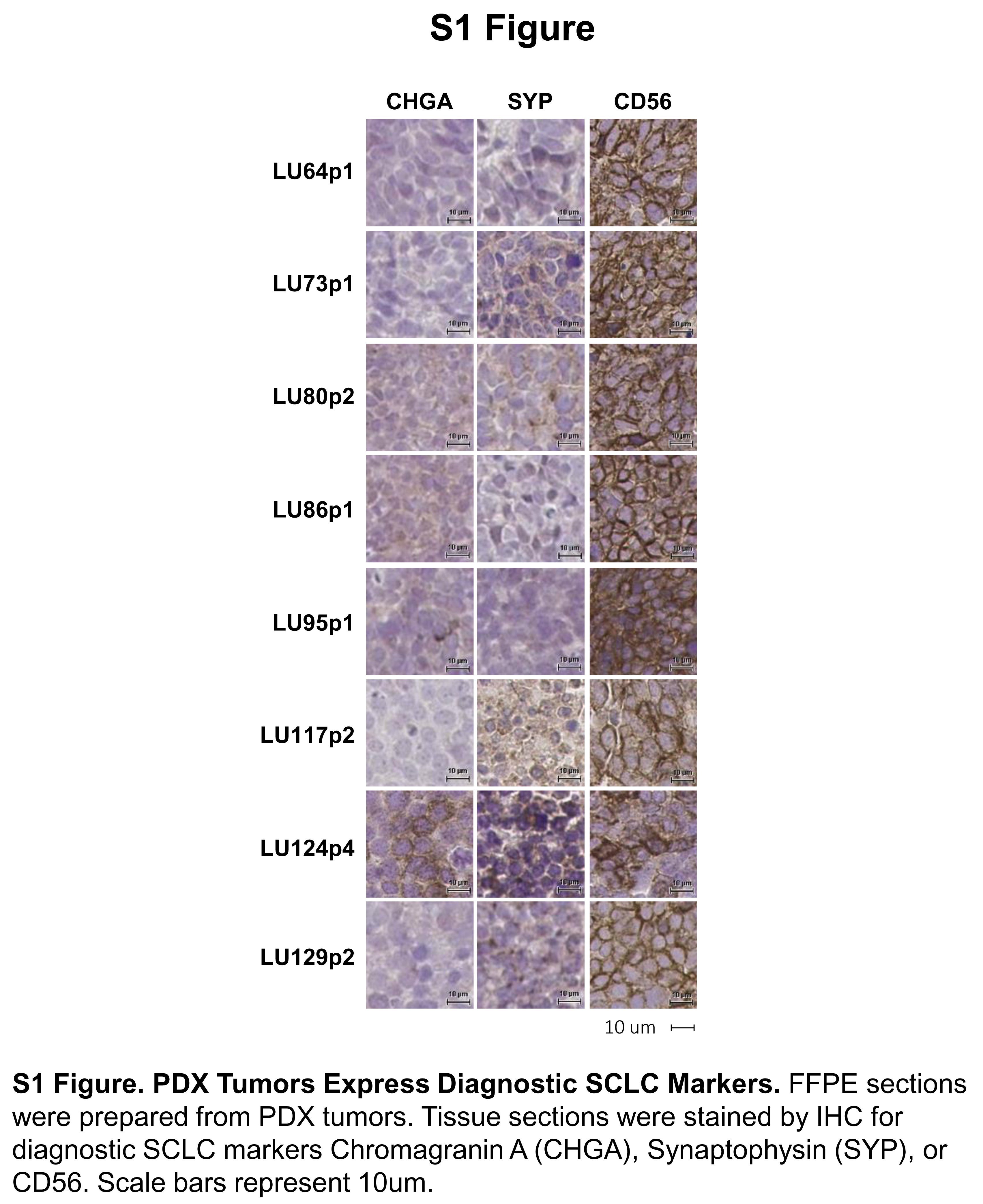

Supplement: S1 Fig — (TIF) [file pone.0125255.s001.tif]

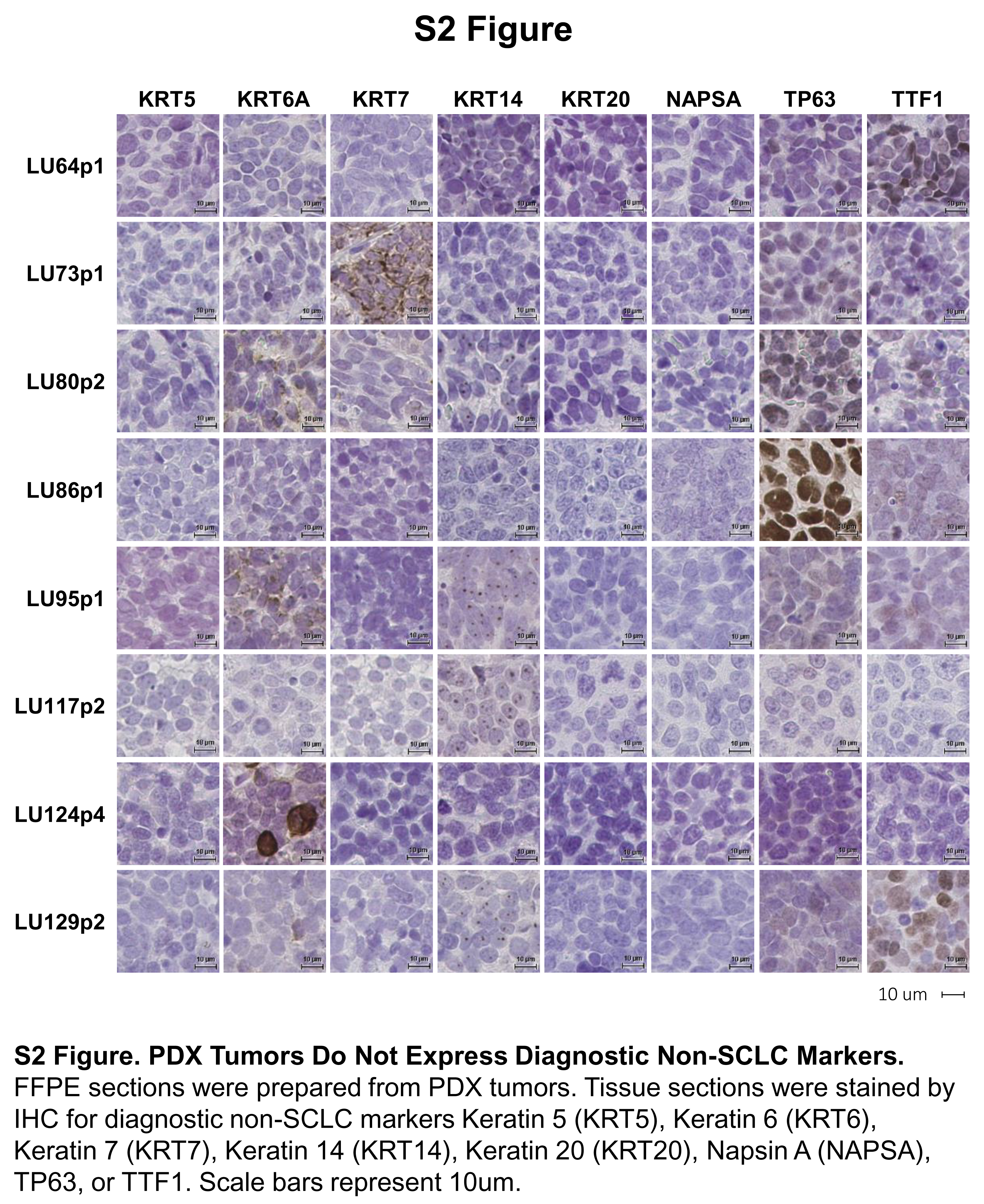

Supplement: S2 Fig — (TIF) [file pone.0125255.s002.tif]

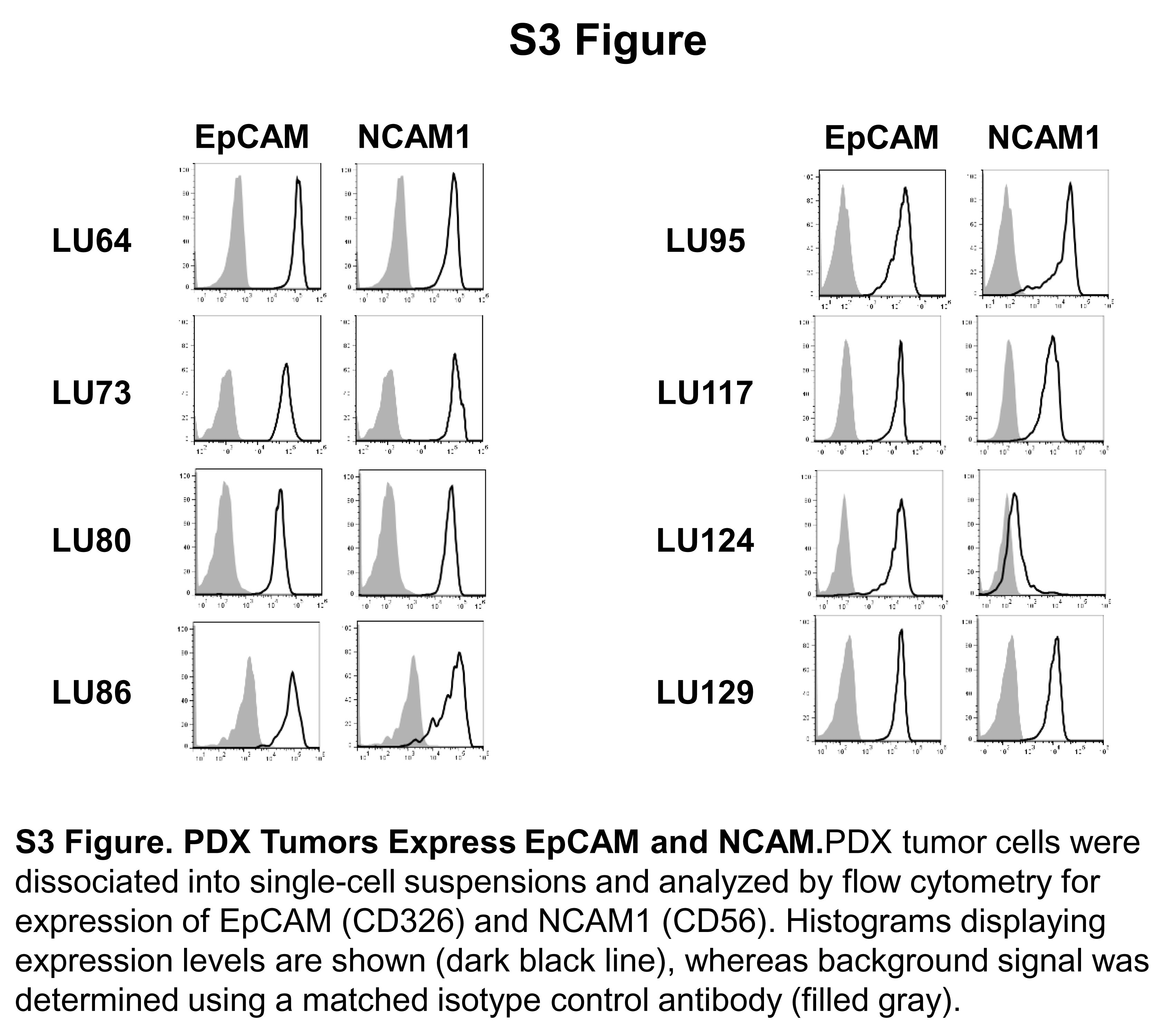

Supplement: S3 Fig — (TIF) [file pone.0125255.s003.tif]

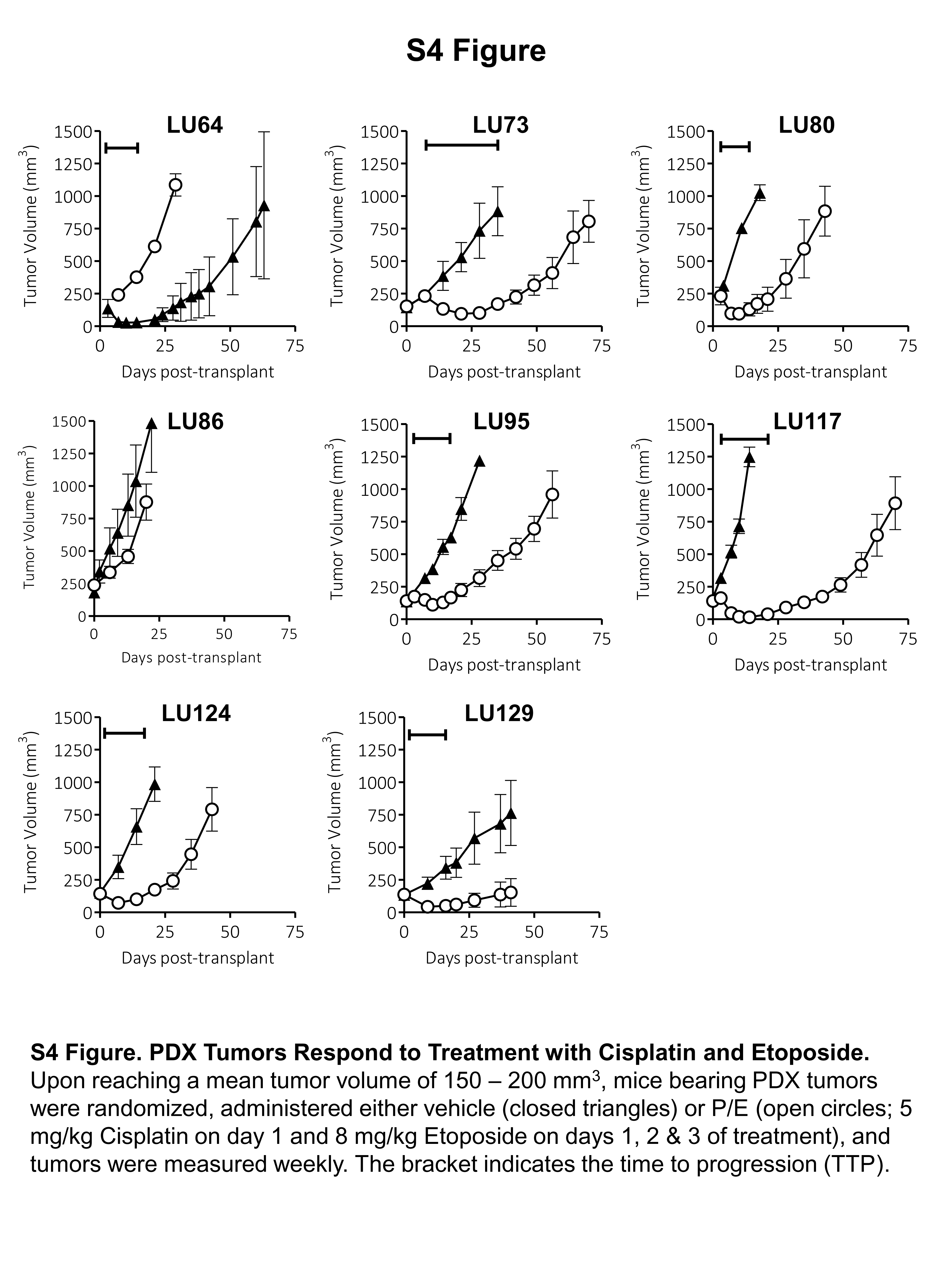

Supplement: S4 Fig — (TIF) [file pone.0125255.s004.tif]

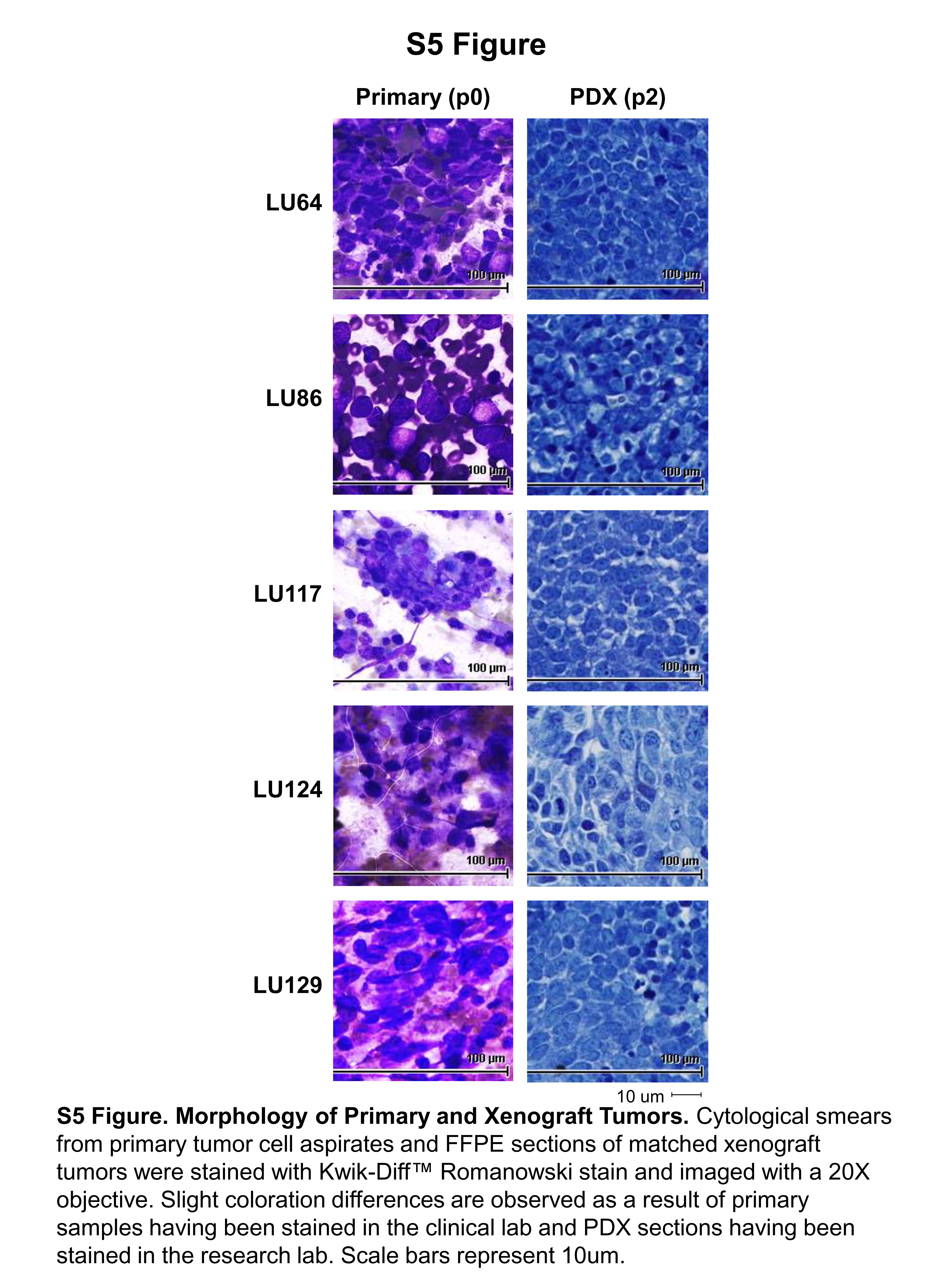

Supplement: S5 Fig — (TIF) [file pone.0125255.s005.tif]

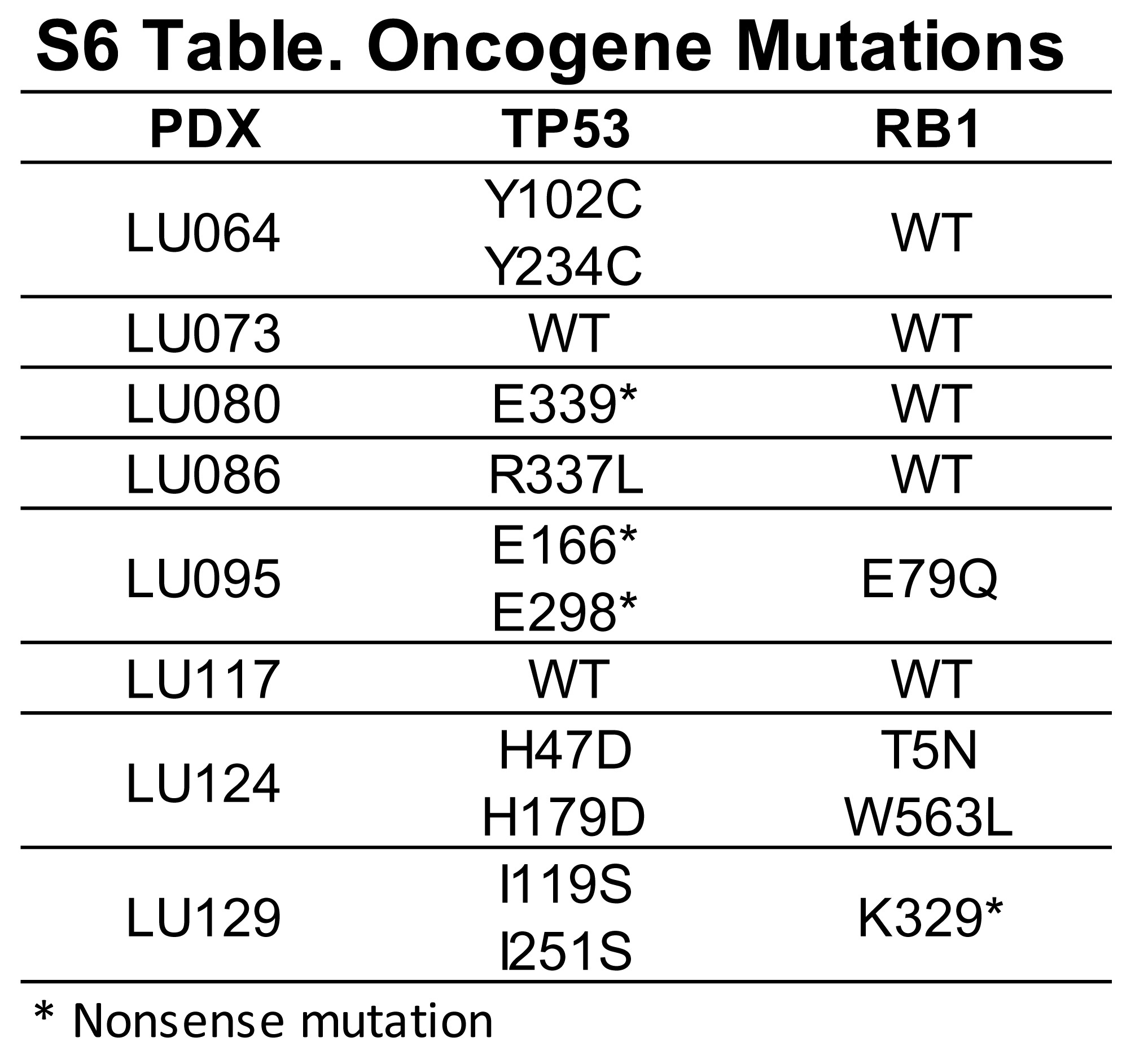

Supplement: S1 Table — (JPG) [file pone.0125255.s006.jpg]
